# Bead-Shaped Mesoporous Alumina Adsorbents for Adsorption of Ammonia

**DOI:** 10.3390/ma13061375

**Published:** 2020-03-18

**Authors:** Jiyull Kim, Hyeonkyeong Lee, Huyen Thanh Vo, Gwoungwoo Lee, Nayeon Kim, Sejin Jang, Ji Bong Joo

**Affiliations:** Department of Chemical Engineering, Konkuk University, 120 Neungdong-ro, Gwangjin-gu, Seoul 05029, Korea; jiyull0630@konkuk.ac.kr (J.K.); hyeonk@konkuk.ac.kr (H.L.); lonbeo2000@gmail.com (H.T.V.); ruddn94@konkuk.ac.kr (G.L.); kny960403@konkuk.ac.kr (N.K.); rkddnr1205@konkuk.ac.kr (S.J.)

**Keywords:** Bead-shaped mesoporous alumina, adsorbent, adsorption, ammonia, chitosan

## Abstract

It is of great importance to remove toxic gases by efficient methods for recovering the atmosphere to safe levels. The adsorption of the toxic gas molecules on solid adsorbents is one of the most useful techniques because of its simple operation and economic feasibility. Here, we report the uniform Bead-Shaped Mesoporous Alumina (BSMA) with tunable particle size for use as an adsorbent for removal of toxic ammonia. The BSMA particles with tunable diameters were synthesized by means of a sol–gel reaction of Al(NO_3_)_3_∙9H_2_O as an alumina precursor in the presence of chitosan as a template. When the ammonia solution is added dropwise to the prepared viscose mixture containing chitosan, acetic acid, and the alumina precursor solution, the sol–gel condensation reaction of the alumina precursor occurs in the chitosan polymer metrics, resulting in bead-shaped chitosan-aluminum hydroxide particles. Then, final Bead-Shaped Mesoporous Alumina (BSMA) particles are obtained by calcination at a high temperature. During the synthesis, changing the mole ratio of the chitosan template to the alumina precursor allowed the particle diameter of the final bead sample to be finely controlled. In addition, the prepared BSMA particles have well-developed mesoporous characteristics with relatively large surface areas, which are beneficial for adsorption of gas molecules. In an ammonia adsorption experiment, the BSMA-1.5 sample, which has the smallest particle diameter among the bead samples, was the best in terms of adsorption capacity. In this manuscript, we systemically discuss the relationship between the characteristics of BSMA samples and their adsorption of ammonia.

## 1. Introduction

There has been increasing attention to the management of atmospheric quality, which can cause severe respiratory health problems for the human body and, occasionally, lead to death, when the atmosphere is significantly polluted by harmful gases [1,2,3]. When toxic gas chemicals are released by a sudden chemical accident, it is important to quickly remove the chemicals and return the atmosphere to safe conditions [4,5]. In modern society, there have been many harmful gases either leaked or exhausted from chemical industries, such as carbon monoxide, hydrogen sulfide, hydrogen chloride, chlorine, phosphine, nitrogen oxide, and ammonia, which should, accordingly, be kept to safe levels [6,7,8,9,10].

Ammonia (NH_3_) is one of the most widely produced inorganic compounds in chemical industries. In addition, it is one of the most useful chemicals in modern society, since it can be used as a fertilizer, precursor to nitrogenous compound, cleaner, fuel, etc [11]. However, it can become a threatening gas that pollutes the atmosphere when too much of it is suddenly exhausted. Since it is colorless, pungent, and corrosive, it can cause fatal damage to humans and animals exposed to it. Although people can smell ammonia without significant symptoms at a level as low as 10 ppm, breathing 50–100 ppm of NH_3_ can give rise to eye, throat, and nose irritation [12]. More severely, prolonged exposure at concentrations higher than 300 ppm can lead to either permanent damage to the body or even death. Therefore, the release of ammonia gas into the atmosphere must be controlled at a suitable level defined by Pollution Prevention and Control (PPC) regulations [13,14].

To remove toxic ammonia molecules, there have been many techniques, including catalytic decomposition, combustion, absorption by solution, and adsorption by solids, employed in not only fundamental studies but also practical processes. Among the above techniques, the adsorption of ammonia on solid adsorbents has been widely studied because of the simple operation of the whole process and the easy regeneration of this valuable gas. To date, various porous materials, including activated carbon, zeolite, alumina, and a metal-organic framework (MOF), have been demonstrated as adsorbents for efficient ammonia removal, and there has been remarkable progress on adsorption of ammonia in terms of either adsorption capacity or adsorption kinetics [5,6,15,16,17,18,19]. Helminen et al. studied the ammonia adsorption capacities of traditional inorganic adsorbents including activated carbon, alumina, silica gel, and zeolite materials. The experimental results indicated that the adsorption capacity values at 97~98 kPa of ammonia conditions are ca. 5, 3, 3.6 and 9 mmol/g for activated carbon, alumina, silica gel, and zeolite-13X, respectively [20]. Saha and co-workers reported that the maximum adsorption of MOF materials at 800 torr of ammonia is about 12.2 mmol/g (207.4 mg/g) [19]. They also investigated ammonia adsorption capacity of porous alumina (3.13 mmol/g, 53.21 mg/g) at 273 K [5]. Kim and co-workers also compared the adsorption capacity of mesoporous alumina, commercial silica, zeolite and activated carbon. The mesoporous alumina showed the highest calculated equilibrium capacity (214 mg/g) of ammonia adsorption at room temperature while other adsorbents, such as commercial silica (33 mg/g), zeolite (43.3 mg/g), and activated carbon (45.8 mg/g) showed relatively low values [18].

In order to achieve high performance of NH_3_ adsorption, adsorbent materials must have favorable physicochemical characteristics, such as large surface area and acidic adsorption sites, since a large amount of basic ammonia should be adsorbed on the surface of adsorbents [21]. Mesoporous alumina, as it has a large surface area, large pore size, and high acidity, should be a candidate as an adsorbent for ammonia removal. Practically, nano-structured porous alumina materials are successfully employed in ammonia adsorption. Saha and co-workers investigated the ammonia-adsorption performance of activated alumina. They synthesized porous alumina and systemically carried out ammonia adsorption [5]. They obtained better adsorption capacity of porous alumina (3.13 mmol/g) and observed that both the porous structure and the acidic surface of alumina are beneficial for the adsorption of base ammonia molecules. Kim et al. also synthesized mesoporous alumina and compared its adsorption capacity with those of other adsorbent materials in a lab-scale adsorbent column [18]. The mesoporous alumina had the highest capacity among the other adsorbents, including commercial silica, zeolite, and activated carbon.

When people developed new adsorbent materials, they tested adsorbents as a fine powder for evaluating their intrinsic adsorption capacity [22], although a practical adsorption column consisted of shaped adsorbent pellets. Even though the powder-type adsorbent has high adsorption capacity, as a result of the advantageous characteristics of the powder itself, such as a large active surface area and short diffusion distance to the adsorption site, it is difficult to directly employ as a bed material in a practical adsorption column, because fine power can be easily lost, leading to a significant pressure drop, which disturbs the normal operation of the whole process [23,24]. Thus, powder-type materials are usually engineered into big pellets (e.g., bead-shaped particles, with a diameter range of μm to mm), since the shaped materials can be easily separated, recycled, and handled [25]. 

In this work, we report on the synthesis of Bead-Shaped Mesoporous Alumina (BSMA) for use as an efficient adsorbent for the removal of ammonia gas. The BSMA particles were synthesized by a template-assisted sol-gel method using chitosan as a template and an alumina precursor. We controlled physical properties, such as the particle diameter of BSMA, by changing synthetic parameters, such as the ratio of the alumina precursor to the template. The prepared BSMA showed a uniform particle size in the range of 0.5~1 mm with well-developed mesoporosity. In ammonia adsorption, the well-optimized BSMA displayed higher uptake capacity for ammonia than commercial gamma alumina. The increased adsorption capacity of BSMA should be attributed to its small particle diameter and well-developed mesoporous characteristics with a large surface area. Based on both the characterization results and ammonia adsorption-performance data, Bead-Shaped Mesoporous Alumina (BSMA) appear to be an ideal adsorbent candidate for toxic-gas removal in a practical adsorption process.

## 2. Materials and Methods 

### 2.1. Materials 

Aluminum nitrate nonahydrate (98%, Al(NO_3_)_3_∙9H_2_O) was purchased from Daejung CHEMICALS & METALS CO.,LTD, (Gyeong-gi, Korea), and used as an alumina precursor. A chitosan, poly(D-glucosamine) (Merck) was used as a template. Acetic acid and ammonium hydroxide (NH_4_OH, 25%) were purchased from Daejung CHEMICALS & METALS CO., LTD, (Gyeong-gi, Korea), and used as received. The commercial gamma alumina nano-powder was purchased from Sigma-Aldrich (St. Louis, MO, USA) and used without further treatment.

### 2.2. Synthesis 

Bead-Shaped Mesoporous Alumina (BSMA) was synthesized by a template-assisted sol–gel reaction of alumina precursor (Al(NO_3_)_3_∙9H_2_O) in the presence of chitosan as a template. Specifically, chitosan polymer (0.015~0.03 mmol) was dissolved in 5% acetic acid (53.9~107.8 mL) in a 250 mL flask. To the above solution, alumina precursor (Al(NO_3_)∙9H_2_O, 0.02 mmol) and D.I. (De-ionized) water solvent (32.6 mL) were added, then the mixture was further stirred for 12 h. Using a syringe pump, the mixture was dropped into a NH_4_OH solution (25 wt.%, 30 mL) at a rate of 1 mL/min under continuous stirring. When the mixture contacted the NH_4_OH solution, spherical precipitates were formed. Finally, Bead-Shaped Mesoporous Alumina (BSMA) was obtained by calcining the spherical precipitates at a desired temperature in air flow for 2 h.

### 2.3. Characterization 

We characterized the particle size and morphology of BSMA by optical microscopy (Super eyes NM-SE02M, NETMATE, Seoul, Korea). The textural properties of samples were characterized by a N_2_ adsorption-desorption technique at 77 K (Micromeritics ASAP 2020, Micromeritics, Norcross, GA, USA, sorption instrument). We estimated the specific surface area of the samples by the Brunauer-Emmett-Teller (BET) formula and obtained pore size distribution by the Barret-Joyner-Halenda (BJH) method using the adsorption branch of isotherms. Crystalline characteristics of the samples were obtained by X-ray diffraction (XRD) using a Rigaku D/mas–220 diffractometer (Rigaku, Japan) with Cu Kα (λ = 1.5418 Ǻ) radiation. We observed the surface morphologies of the BSMA by scanning electron microscopy (SEM, JSM-6060, JEOL, Tokyo, Japan). The TGA experiment was carried out using a Bruker TG-DTA 2000 instrument (Bruker, Billerica, MA, USA).

### 2.4. Adsorption Experiment 

For the ammonia adsorption, NH_3_ (5000 ppm balanced with N_2_) and dried N_2_ (99.999%) were used as an adsorbate gas and a carrier gas, respectively. Adsorption experiments were conducted by using a home-made adsorption system using a fixed-bed adsorbent column [18]. The experimental apparatus consisted of a stainless-steel adsorption column with adsorbent bed, mass-flow controller, six-port valve connected with a sample loop, and an ammonia gas detector (GAS TIGER 6000, Wamdi; China) for detecting the outlet NH_3_ concentration. Before conducting the adsorption experiment, we pretreated the adsorbent placed in the adsorption column in a N_2_ flow at 300 °C to remove any residual water and organic contaminants. Then, the adsorption column was cooled to room temperature (25 °C). In order to measure the adsorption capacity and kinetics, ammonia was purged into the reaction system and the adsorption experiment was started. The concentration of the outlet ammonia was measured every 10 mins. The adsorption would be finished when the detected concentration of the outlet gas was equal to the detected value of the inlet gas (5000 ppm).

## 3. Results and Discussion

**Figure 1:**
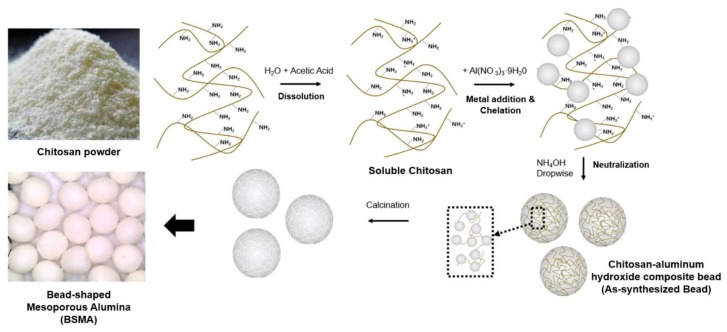
Schematic illustration for the synthesis of Bead-shaped Mesoporous Alumina (BSMA) adsorbent.

Bead-Shaped Mesoporous Alumina (BSMA) was synthesized by a biopolymer template-assisted sol–gel reaction of alumina precursor followed by sequential calcination at 600 °C (Figure 1) [26,27,28]. 

More specifically, the synthesis of BSMA consisted of the following steps. First, chitosan as the template for the formation of bead-shaped porous particles was dissolved in the diluted acetic-acid solution [29]. Since the chitosan has a pK_a_ value of ca. ~6.5, the diluted acetic-acid solution helps chitosan to be easily water soluble [30,31]. Then, the alumina precursor (Al(NO_3_)_3_∙9H_2_O) was added to the above mixture under vigorous stirring. During this step, the amine functional group of chitosan could interact strongly with Al^3+^ ions, resulting in chelation of the aluminum ion-amine in the polymer network of chitosan [29,32]. When the above mixture solution was slowly added dropwise to the diluted ammonia solution, the amino functional group of chitosan was neutralized, and the soluble chitosan was re-precipitated as a stable bead shape in the solution [29,30,33]. At the same time, the alumina precursor was hydrolyzed and condensed in the polymer matrix of chitosan beads, resulting in a chitosan-aluminum hydroxide composite. Finally, the Bead-Shaped Mesoporous Alumina (BSMA) was obtained by calcining the as-synthesized chitosan-aluminum hydroxide composite beads (as-synthesized beads) at 600 °C. Since the chitosan template plays an important role not only in formation of the bead-shaped particles but also as a pore-forming agent, BSMA can be obtained by removing the chitosan by calcination at a high temperature.

**Figure 2:**
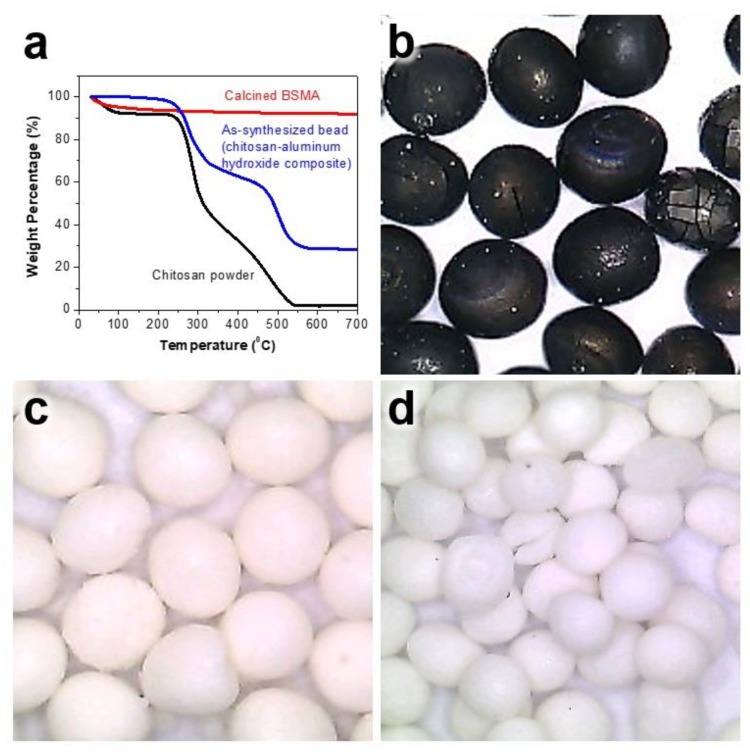
(**a**) TGA results of chitosan powder as a reference, as-synthesized bead and calcined BSMA sample at 600 °C. Digital photo images of BSMA samples calcined at various temperature: (**b**) 400 °C, (**c**) 600 °C and (**d**) 800 °C, respectively.

Since the as-synthesized beads not only consisted of the chitosan template but also had an amorphous phase, heat treatment is necessary to remove chitosan for generating mesopores as well as to induce crystallization of the amorphous alumina to its metastable gamma counterpart. To find the calcination temperature of BSMA materials, we used TGA analysis. Figure 2a shows the TGA results for the template chitosan powder as a reference run, as-synthesized bead (chitosan-aluminum hydroxide), and the calcined BSMA at 600 °C. 

In the TGA data of the chitosan powder, it displays weight loss in the range of ambient temperature to ca. 100 °C, which is attributed to evaporation of weakly interacting water molecules. The TGA curve of chitosan displays a significant weight loss in the range of 250 to 320 °C, indicating the thermal decomposition of the weak functional group in the chitosan molecule. In the temperature range of 320 to 580 °C, the chitosan shows continuous weight loss, meaning thermal oxidation and decomposition of chitosan, which is completely burned out after 600 °C. The as-synthesized bead displays a TGA curve change in the temperature range of 250 to 320 °C, indicating that the weak functional group in the chitosan molecule was decomposed. In addition, it also has a gradual decrease followed by a dramatic weight loss in the range of 320 to 580 °C, indicating that the chitosan molecules incorporated in the as-synthesized chitosan-aluminum hydroxide bead were continually burned out and decomposed. However, the BSMA sample calcined at 600 °C shows different thermogravimetric behavior. It does not display any major weight loss in any temperature range, indicating there are no obvious combustible carbon species. 

We also used optical microscopy to confirm the suitable calcination temperature and morphology of the final BSMA samples. As shown in Figure 2b, even though the BSMA sample prepared by calcination at 400 °C displayed a uniform spherical morphology, the particles were black, indicating the presence of unburned carbon species originating from incomplete combustion of chitosan at too low a calcination temperature [34,35]. As the calcination temperature increased to 600 and 800 °C, the final BSMA sample was white, indicating that the chitosan template is completely combusted, leaving a pure alumina species (Figure 2c,d). In addition, even though the BSMA sample was treated at 800 °C, the spherical morphology was well maintained, indicating the thermal stability of the BSMA sample. Based on the above results, as-synthesized chitosan-aluminum hydroxide beads should be calcined at above 600 °C.

**Figure 3:**
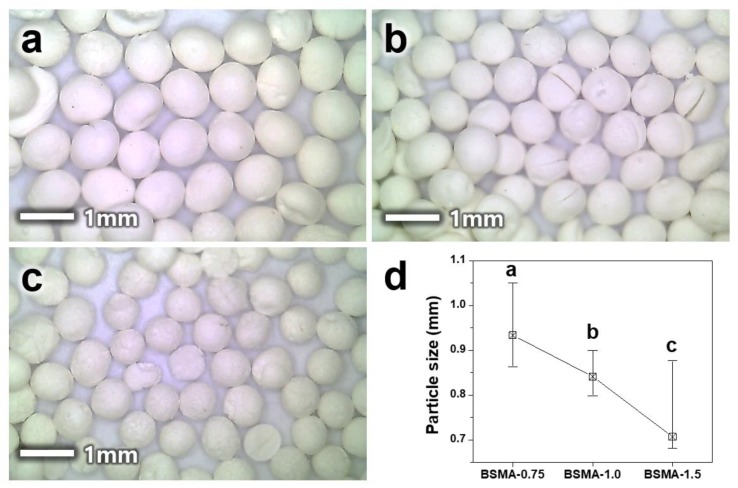
Digital photo images of BSMA samples prepared by using different mole ratio of chitosan to alumina precursor: (**a**) 0.75:1 (BSMA-0.75), (**b**) 1:1 (BSMA-1.0) and (**c**) 1.5:1 (BSMA-1.5). (**d**) Average particle size of each BSMA samples.

Consistent with other syntheses, synthesizing BSMA particles were controlled by varying the synthetic parameters. We controlled the size of the BSMA particles by changing the ratio of template and alumina precursor. As shown in Figure 3a, when the mole ratio of chitosan to alumina precursor is 0.75:1 in the synthesis followed by calcination at 600 °C, the BSMA-0.75 particle was a uniform sphere with an average diameter of ca. 0.92 mm. 

When the ratio of chitosan to the alumina precursor changed to 1:1 and 1.5:1, the particle diameter of the final BSMA was slightly decreased, to ca. 0.84 and 0.77 mm, respectively (Figure 3b,c). As shown in Figure 3d, the average particle diameter of BSMA continuously decreased, from 0.92 to 0.84, and 0.77 mm, with the change in the ratio of the chitosan template to the alumina precursor from 0.75:1 to 1:1, and 1.5:1, respectively. In order to estimate uniformity and average particle size, we counted more than 60 particles from digital photo images and visualized particle size as a histogram (Figure S1, Supporting Information). Although each sample does not show a perfect symmetry of the Gaussian distribution curve, we can determine center peaks using the particle size distribution. BSMA-0.75 has a distribution with the peak position at the largest particle size (0.92 mm) compared to other samples. When the ratio of chitosan to the alumina precursor changed to 1:1 and 1.5:1, the distributions shifted to the left, indicating the smaller size. BSMA-1.0 and BSMA-1.5 have the center peaks at 0.84 and 0.74 mm (Figure S1). 

As reported in a previous study, the polymer surfactants that contain many hydroxy and other functional groups can generate strong binding with the surface of metal-oxide particles, such as SiO_2_, TiO_2_, or Al_2_O_3_. In addition, the polymer surfactant can have a strong interaction with the hydrolyzed alumina precursor, resulting in the controlled depositing onto the surface of the alumina particles. Although it is well known that a high concentration of polymer surfactant induces controlled growth of the particles, it suppresses the growth rate of particles and interparticle aggregation, resulting in small, uniform particles. In our work, chitosan is a well-known biopolymer surfactant, and it should play a similar role in synthesis. Both hydroxy and amine functional groups should strongly interact on both the alumina surface and the alumina precursor. The increased chitosan concentration induces more suppression on particle growth, in which it induces a decrease of particle size.

**Figure 4:**
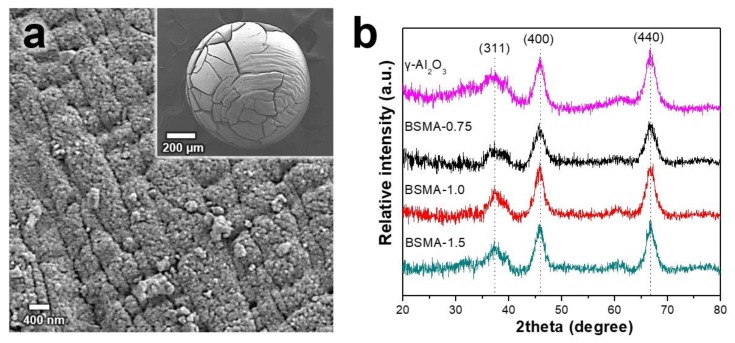
(**a**) SEM image of BSMA-1.5 and (**b**) XRD patterns of commercial γ-Al_2_O_3_ and BSMA samples prepared by using different mole ratios of chitosan to alumina precursor.

We investigated the surface characteristics and crystalline properties of the prepared BSMA samples by SEM and XRD. As shown in Figure 4a, the SEM images clearly show spherical bead particles with a diameter of ca. 0.8 mm. 

They show an obvious crack in the thick alumina layer, with plenty of small pores and a rough surface, indicating a well-developed porous structure. Figure 4b shows XRD patterns of commercial γ-Al_2_O_3_ and BSMA samples prepared with different mole ratios of the chitosan template to the alumina precursor. Commercial γ-Al_2_O_3_ and all BSMA samples revealed typical diffraction pattens of the gamma alumina phase at 2θ = (37.6°, 45.8°, and 66.8°) attributed to (311), (400) and (440), respectively [36]. Interestingly, commercial γ-Al_2_O_3_ and all BSMA samples showed similar peak sharpness, indicating similar crystallinity. Although the BSMA samples prepared with different mole ratios of the chitosan template to the alumina precursor have different particle sizes, they have similar crystalline properties, because all the samples are calcined at identical temperature (e.g., 600 °C).

**Figure 5:**
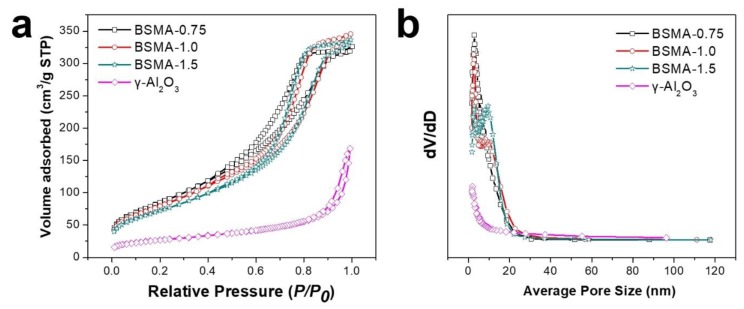
(**a**) N_2_ isotherm and (**b**) BJH pore size distribution of commercial γ-Al_2_O_3_ and BSMA samples prepared by using different mole ratio of chitosan to alumina precursor.

We also investigated the textural characteristics of commercial γ-Al_2_O_3_ and the BSMA samples prepared with different mole ratios of the chitosan template to the alumina precursor by using a nitrogen adsorption-desorption technique. Figure 5 provides the nitrogen adsorption-desorption isotherm and corresponding Barrett–Joyner–Halenda (BJH) pore-size distributions of the BSMA samples. 

As shown in Figure 5a, commercial γ-Al_2_O_3_ exhibited a type-II-like isotherm with steep increase of adsorption capacity in the range of high relative pressure (P/P_0_ > 0.9). It indicates nonporous characteristics of alumina nanoparticulate. As shown in Figure S2 (Supporting Information), the commercial γ-Al_2_O_3_ exhibited the aggregated form of nonporous alumina nanoparticles. Although it showed big interparticle spaces, they cannot contribute to the porosity of the sample. The commercial γ-Al_2_O_3_ had the lowest capacity in the range of monolayer adsorption (0.1 < P/P_0_ < 0.25) among the alumina samples, indicating the smallest BET surface area value (93 m^2^/g, Table S1). BSMA-0.75 displayed a representative type-IV isotherm with a well-developed hysteresis loop, indicating the well-developed mesoporosity [37]. The BSMA-0.75 sample had a relatively large capacity in the range of monolayer adsorption (0.1 < P/P_0_ < 0.25), indicating a large BET surface area. Practically, the specific surface area of BSMA-0.75 is estimated to be ca. 317 m^2^/g. In addition, it shows a steep increase of adsorption in the pressure range of 0.4~0.8, indicating the presence of uniform, well-developed mesopores. Other BSMA-1 and BSMA-1.5 samples showed similar patterns of the nitrogen adsorption isotherm. Both BSMA-1 and BSMA-1.5 samples showed a type-IV isotherm with a well-developed hysteresis loop. Although BSMA-1.5 showed a slightly lower adsorption capacity in the pressure range of 0.1~0.25, the BSMA-1 sample exhibited a similar adsorption capacity in the same range compared to BSMA-0.75. Both BSMA-1 and BSMA-1.5 samples showed a similar hysteresis loop in the pressure range of 0.4~0.8, indicating similar pore-size distributions. The specific surface areas of BSMA-1 and BSMA-1.5 were 297 and 267 m^2^/g, respectively. As shown in Figure 5b and Table S1, the commercial γ-Al_2_O_3_ does not exhibit obvious pore size distribution in the meso-macro pore range, although it has a small volume increase in the range of <2 nm, indicating nonporous characteristics of alumina nanoparticulate. However, all the BSMA samples displayed obvious peaks in the range of 1~20 nm, which indicated the presence of a major mesopore with minor micropores. Based on the above characterization results, Bead-Shaped Mesoporous Alumina (BSMA) particles were successfully synthesized using chitosan as a template and an alumina precursor. By varying the mole ratio of the chitosan template to the alumina precursor, the particle diameter can be controlled, even though the textural characteristics of the BSMA samples are negligibly changed.

**Figure 6:**
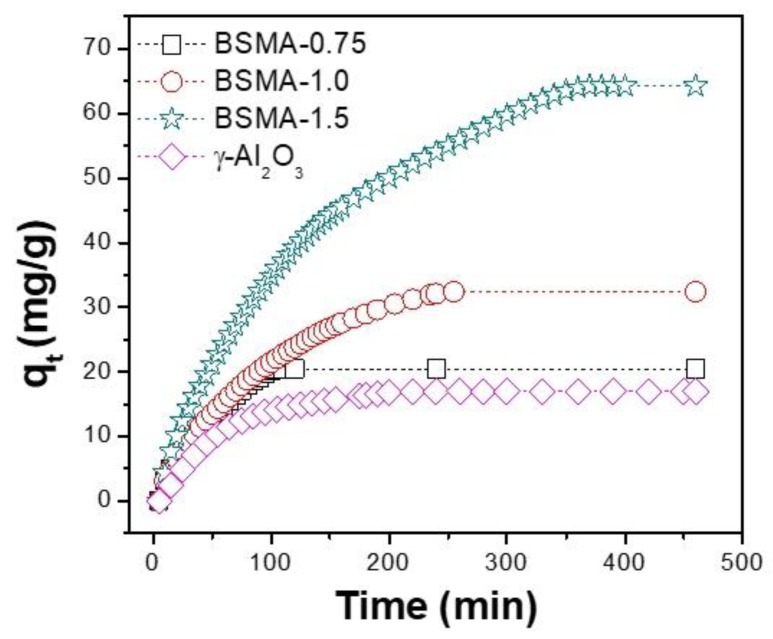
Adsorption performance results of commercial gamma alumina and the synthesized BSMA samples.

The prepared BSMA samples were used as adsorbents for gas phase ammonia adsorption. To compare the adsorption performance with that of other commercial materials, the commercial alumina nanopowder (Sigma-Aldrich, St. Louis, MO, USA; surface area is 93 m^2^/g) with the γ-alumina phase was used as a reference adsorbent. The adsorption curves of BSMA and commercial γ-alumina were collected at ambient conditions using a simulated ammonia gas flow with 5000 ppm NH_3_ in nitrogen. As shown in Figure 6, the commercial alumina showed relatively small adsorption capacity (ca. 17 mg/g). 

The prepared BSMA samples have larger capacities for ammonia adsorption in the range of 22.5~66.2 mg/g than commercial alumina does. The measured adsorption capacity for BSMA-0.75, BSMA-1.0, and BSMA-1.5 were 22.5, 34.1, and 66.2 mg/g, respectively. In particular, the BSMA-1.5 sample showed the largest adsorption capacity during a prolonged adsorption time (~450 min). The adsorption of ammonia over BSAM-1.0 and BSMA-0.75 is saturated within ca. 230 and 120 min, respectively.

**Figure 7:**
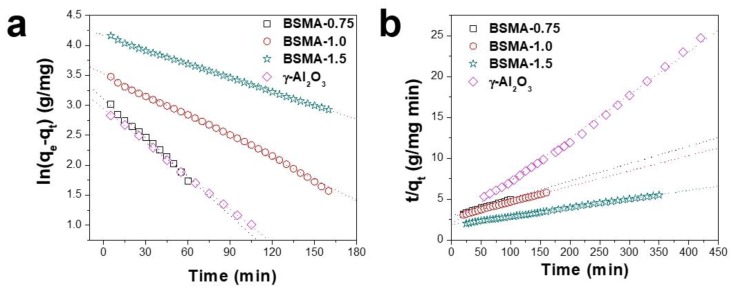
(**a**) Pseudo-first-order and (**b**) pseudo-second-order kinetic plots of commercial gamma alumina and the synthesized BSMA adsorbents on ammonia adsorption.

In order to compare adsorption kinetics, we replotted the previous adsorption data to fit both a pseudo-first-order adsorption kinetic formula and a pseudo-second-order adsorption kinetic one (Figure 7). 

The pseudo-first-order adsorption kinetic formula is as follows [38,39]:

(1) $$\frac{dq_{t}}{dt}=k_{1}\left(q_{e}-q_{t}\right)$$

(2) $$\ln\left(q_{e}-q_{t}\right)=\ln q_{e}-k_{1}t$$

where *k*_1_ is the adsorption kinetic constant (1/min), *q_t_* is the adsorption amount (mg/g) at time *t*, and *q_e_* is adsorption capacity (mg/g) at equilibrium.

The pseudo-second-order adsorption kinetic formula is as follows:

(3) $$\frac{dq_{t}}{dt}=k_{1}{\left(q_{e}-q_{t}\right)}^{2}$$

(4) $$\frac{t}{q_{t}}=\frac{1}{k_{2}q_{e}^{2}}+\frac{1}{q_{e}}t$$

where *k*_2_ is the adsorption kinetic constant (g/mg∙min), *q_t_* is the adsorption amount (mg/g) at time *t*, and *q_e_* is the adsorption capacity (mg/g) at equilibrium. 

Table 1 summarizes the adsorption kinetic constant of each sample and the coefficient of determination (r^2^). In the first-order adsorption kinetic model, the coefficient of determination (r^2^) of the BSMA samples is in the range of 0.994~0.999. The BSMA samples also showed a high coefficient of determination (r^2^ = 0.996~0.999) in the second-order model. Both the first-order and second-order adsorption kinetic models showed a high coefficient of determination, indicating that both adsorption kinetics models are well fit over the BSMA samples. BSMA-0.75 showed the largest kinetic constant values (k_1_ = 0.02186 min^−1^ and k_2_ = 0.000867 g/mg∙min) among the samples tested. BSMA-1.5 sample, which had the highest adsorption capacity, exhibited the lowest rate constant values (k_1_ = 0.00776 min^−1^ and k_2_ = 0.000135 g/mg∙min). The kinetic constant values of the alumina samples for ammonia adsorption followed the order: BSMA-0.75 ≥ commercial Alumina > BSMA-1.0 > BSMA-1.5.

It is generally known that ammonia adsorption on the alumina surface is originated from acid–base interactions between base ammonia adsorbate and acidic surface. In other words, the chemical characteristics and surface properties of alumina adsorbents significantly influenced the adsorption performance [5,40,41]. Since ammonia molecules can be adsorbed on both Bronsted acidic centers (Al-O^−^) and Lewis acidic sites (Al^(+)^) of the alumina surface, it is important to control both acidic centers to achieve enhanced adsorption performance. 

However, the adsorption capacity and kinetics of the BSMA samples can be explained by differences in the physical properties of adsorbents rather than chemical properties. Based on the characterization results, BSMA-x samples were synthesized by varying the mole ratio of chitosan template to alumina precursor, and the particle size became smaller with an increased ratio of chitosan. Since all BSMA samples were calcined at the same temperature (600 °C), they exhibited similar crystalline properties and surface acidity properties. Even though BSMA-1.5 particles showed a slightly smaller specific surface area, all the BSMA samples showed similar textural properties, including surface area and pore-size distributions. In ammonia adsorption, BSMA-1.5, which is the smallest particle, showed the largest ammonia-adsorption capacity. Since the physicochemical properties of the BSMA samples were similar except for the particle diameter, it should be noted that the performance differences are originated from the different particle sizes of the BSMA samples. 

In adsorption phenomena, the adsorbate molecules must be diffused into the adsorption site of the porous adsorbent. During the limited residence time, adsorption first occurs on the outer surface of the BSMA particle and then ammonia molecules penetrate into the interior, inducing adsorption into the bulk area of the particle. Since the size of a BSMA particle is on the mm scale, the diffusion length must be extremely large to use all of the adsorption sites, compared to the size of the adsorbate ammonia molecules (Ǻ). The effective diffusion length should be limited in the limited residence time by a huge difference between adsorbent size (mm) and adsorbate molecule dimension (Ǻ). Thus, when the adsorbate molecule can penetrate a similar shell length of BSMA particles, adsorption should happen at a similar length from the outer surface. As particle sizes become smaller, the number of particles employed in the adsorption column is increased, indicating more particles with a similar diffusion region during the adsorption process. Therefore, the BSMA-1.5 sample should have more chance to provide a very effective adsorption site in the limited residence time, resulting in large adsorption capacity; however, it takes a long time for the adsorption sites to be saturated, inducing low adsorption kinetics. In contrast, the number of particles employed in the adsorption column is relatively small in the case of the BSMA-0.75 sample, which has the biggest particle size. BSMA-0.75 should provide a relatively small number of adsorption sites with a similar diffusion length, resulting in small adsorption capacity and it showed fast kinetics on saturation of adsorption sites. As shown in Figure S3, the experimental adsorption capacity is decreased from 20.4 to 64.2 mg/g, with an increase of BSMA size from 0.74 to 0.93 mm. In contrast, as the size of particle increased, the peudo-first-order kinetic constant of each BSMA sample increased from 0.00776 to 0.02186 min^−1^. 

## 4. Conclusions

We synthesized the uniform Bead-Shaped Mesoporous Alumina (BSMA) with particle sizes and well-developed mesoporosity for use as an efficient adsorbent for ammonia adsorption. The synthesis involves several sequential processes: (i) Dissolution of the chitosan template in the diluted acetic-acid solution; (ii) chelation between the aluminum ion and the amine functional group in the polymer network of the chitosan by adding the alumina precursor to the aqueous chitosan solution; (iii) Formation of a bead-shaped chitosan-aluminum hydroxide composite by neutralization using diluted ammonia, and (iv) calcination of the composites to obtain BSMA. The particle size of the final BSMA samples can be feasibly tuned by varying the mole ratio of chitosan to alumina precursor, which significantly influences the adsorption performance. In particular, the BSMA-1.5 adsorbent, which has the smallest particle diameter, has the best adsorption capacity. Since the size of a BSMA particle (mm scale) is big enough, compared to the size of ammonia molecules (Ǻ), adsorption should happen in a similar length of the outer layer in the limited residence time. Among the alumina adsorbent samples, BSMA-1.5, with the smallest particle size, has more chance to use adsorption sites effectively, resulting in a large adsorption capacity. We believe that our BSMA particle should be a good candidate as an adsorbent for the removal of toxic gas in a practical adsorption column process.

## Figures and Tables

**Table 1:** Pseudo-first-order and pseudo-second-order kinetic model parameters of commercial gamma alumina and the synthesized BSMA adsorbents on ammonia adsorption.

Sample	Experimental Equilibrium Capacity q_e,exp_ (mg/g)	Pseudo First Order Kinetic Model			Pseudo Second Order Kinetic Model		
		Calculated Equilibrium Capacity q_e,cal_	k_1_ (1/min)	r^2^	Calculated Equilibrium Capacity q_e,cal_	k_2_ (g/mg min)	r^2^
BSMA-0.75	20.4	21.71	0.02186	0.9945	46.9	0.000867	0.99812
BSMA-1.0	32.3	33.54	0.0117	0.9961	52.1	0.000352	0.9992
BSMA-1.5	64.2	63.37	0.00776	0.9992	93.0	0.000135	0.9999
γ-Al_2_O_3_	16.9	18.46	0.01862	0.9990	18.7	0.002081	0.9964

## Supplementary Materials

The following are available online at https://www.mdpi.com/1996-1944/13/6/1375/s1, Figure S1: Particle size distribution histogram of each BSMA samples, Figure S2: TEM image of commercial γ-Al_2_O_3_ sample, Figure S3: The relationship between equilibrium adsorption capacity, peudo-first order kinetic constant and the particle size of BSMA samples, Table S1: Pore Properties, particle size and NH_3_ adsorption capacity of the alumina samples used in this work.
