# Supplementary material for: Bead-Shaped Mesoporous Alumina Adsorbents for Adsorption of Ammonia
Source: Materials (Basel). 2020 Mar 18;13(6):1375. doi: 10.3390/ma13061375 (PMC7143451; doi:10.3390/ma13061375)
Supplement: Supplementary file 1 [file materials-13-01375-s001.pdf]

materials

Supporting Information

Bead-Shaped Mesoporous Alumina Adsorbents for Adsorption of Ammonia

Jiyull Kim, Hyeonkyeong Lee, Huyen Thanh Vo, Gwoungwoo Lee, Nayeon Kim, Sejin Jang and Ji Bong Joo *

Department of Chemical Engineering, Konkuk University, 120 Neungdong-ro, Gwangjin-gu, Seoul 05029, Korea; jiyull0630@konkuk.ac.kr (J.K.), hyeonk@konkuk.ac.kr (H.L.) lonbeo2000@gmail.com (H.T.V.), ruddn94@konkuk.ac.kr (G.L.), kny960403@konkuk.ac.kr (N.K.), rkddnr1205@konkuk.ac.kr (S.J.)

* Correspondence: jbjoo@konkuk.ac.kr; Tel.: +82-2-450-3545

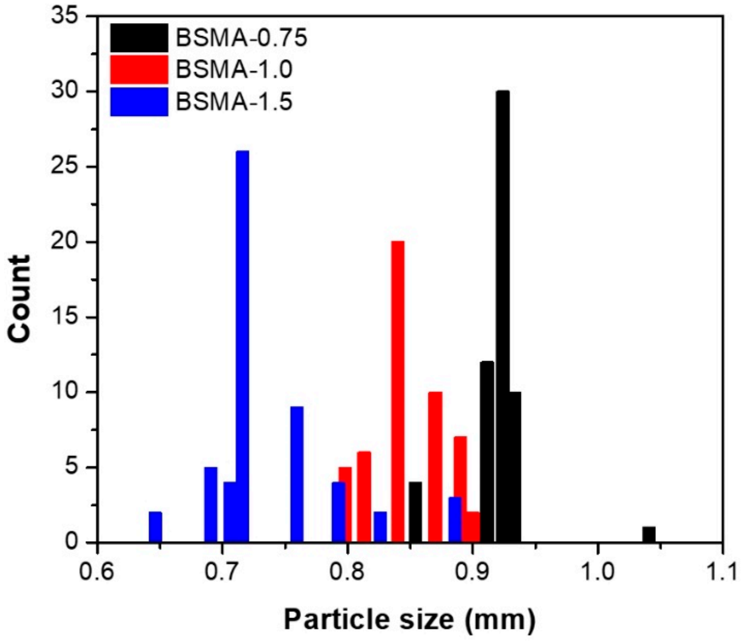

Figure S1. Particle size distribution histogram of each BSMA samples.

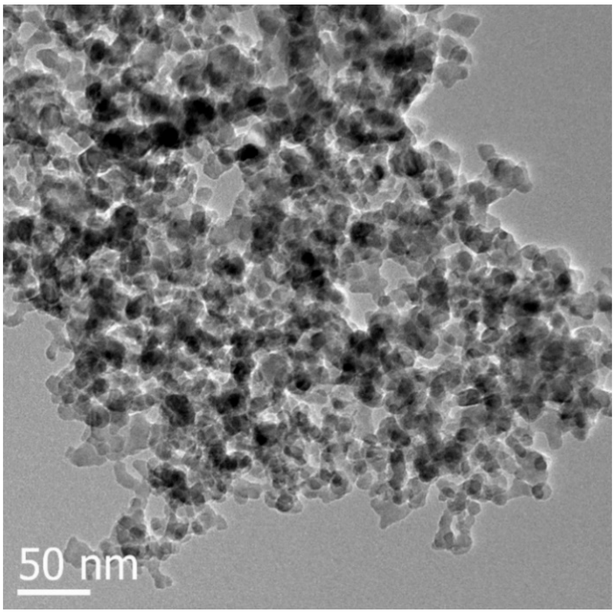

Figure S2. TEM image of commercial γ-Al_2O_3 sample.

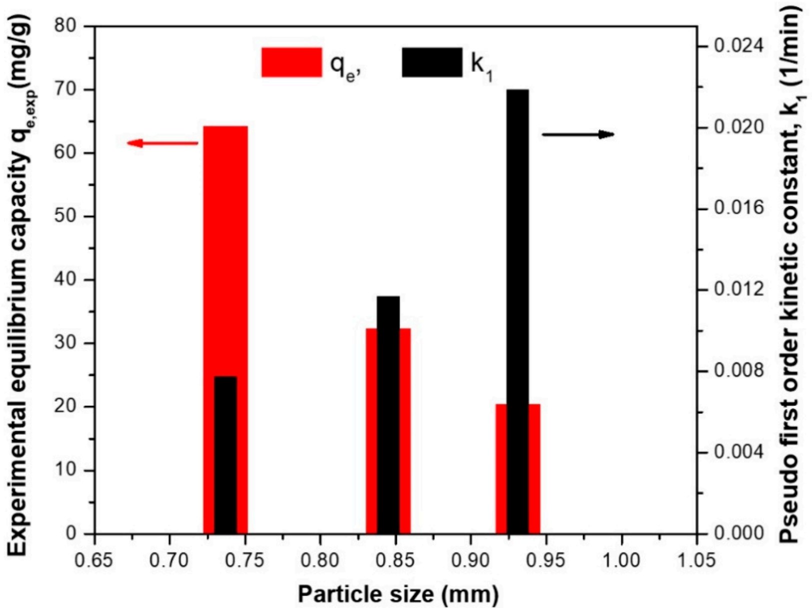

Figure S3. The relationship between equilibrium adsorption capacity, peudo-first order kinetic constant and the particle size of BSMA samples.

Table S1. Pore Properties, particle size and NH_3 adsorption capacity of the alumina samples used in this work.

Sample	Specific Surface Area (m^2/g)	Calculated Pore Size (nm)	Pore Volume (cm^3/g)	Particle Size (mm)	NH_3 Adsorption Capacity (mg/g)
BSMA-0.75	317	6.19	0.4912	0.74	20.4
BSMA-1.0	297	6.94	0.5180	0.84	32.3
BSMA-1.5	267	7.60	0.5085	0.93	64.2
γ- Al_2O_3	93	7.77*	0.1859*	-	16.9

*Since γ-Al_2O_3 have not porous nanostructure, its pore size and pore volume in the table mean the void fraction originated from aggregation between primary particles.

© 2020 by the authors. Licensee MDPI, Basel, Switzerland. This article is an open access article distributed under the terms and conditions of the Creative Commons Attribution (CC BY) license (http://creativecommons.org/licenses/by/4.0/).
